# Racial disparities in receipt of standard chemoradiation in anal squamous cell carcinoma, an analysis of the National Cancer Database

**DOI:** 10.1002/cam4.3625

**Published:** 2020-12-11

**Authors:** Shelly X. Bian, Dennis H. Chen, Eugene Lin

**Affiliations:** ^1^ Department of Radiation Oncology Keck School of Medicine University of Southern California Los Angeles CA USA; ^2^ Department of Medicine Division of Nephrology University of Southern California Los Angeles CA USA; ^3^ Leonard D Schaeffer Center for Health Policy and Economics University of Southern California Los Angeles CA USA

**Keywords:** anal, cancer, chemoradiation, disparity, race, squamous

## Abstract

**Background:**

Standard treatment for locally advanced anal squamous cell carcinoma (SCC) consists of concurrent chemoradiation. We evaluated whether racial differences exist in the receipt of standard treatment and its association with survival.

**Methods:**

From the National Cancer Database, we identified patients diagnosed with anal SCC (Stages 2–3) between 2004 and 2015. Using logistic regression, we evaluated racial differences in the probability of receiving standard chemoradiation. We used Cox proportional hazards models to evaluate associations between race, receipt of standard therapy and survival.

**Results:**

Our analysis included 19,835 patients. Patients receiving standard chemoradiation had better survival than patients receiving nonstandard therapy (hazard ratio [HR] 0.64; 95% confidence interval [CI] 0.61–0.68; *p* < 0.001). Compared to White patients, Black patients were less likely to receive standard therapy (odds ratio [OR] 0.85; 95% CI 0.76–0.96; *p* < 0.008). We observed no statistical difference in mortality between Black and White patients overall (HR 1.05, 95% CI 0.97–1.15; *p* = 0.24). However, for the subgroup of patients receiving nonstandard therapy, Black patients had an increased mortality risk compared to White patients (HR 1.17, CI 1.01–1.35; *p* = 0.034). We observed no survival differences in the subgroup of patients receiving standard treatment (HR 1.00, CI 0.90–1.11, *p* = 0.99).

**Conclusion:**

Standard treatment in anal SCC is associated with better survival, but Black patients are less likely to receive standard treatment than White patients. Although Black patients had higher mortality than White patients in the subgroup of patients receiving nonstandard therapy, this difference was ameliorated in the subset receiving standard therapy.

## Graphical abstract

Black patients are less likely than White patients to receive standard chemoradiation, which is associated with improved survival. While no racial differences in survival were observed overall, there was significantly lower survival in Black versus White patients who did not receive standard therapy.

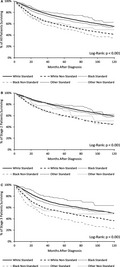

## 1 INTRODUCTION

Anal cancer represents an estimated 2.5% of all GI malignancies in the United States,^1^ the majority of which are squamous cell carcinomas (SCC).^2^ Though relatively rare, the incidence of anal cancer has steadily increased over the last 40 years.^3^, ^4^ Historically, abdominoperineal resection (APR), which includes a complete resection of the anal sphincter complex, was the mainstay of treatment. In 1983, Nigro et al. found that chemoradiation therapy with 5FU and mitomycin C (MMC) induced a complete response in 22 of 28 patients, sparing patients of a permanent colostomy.^5^

Multiple randomized clinical trials have since established concurrent chemoradiation therapy as the most effective initial treatment modality for nonmetastatic anal SCC, reserving APR for salvage.^6^ The ACT I Trial in 1996 demonstrated superiority of concurrent chemoradiation with mitomycin and 5FU to radiation alone in terms of locoregional recurrence and relapse free survival.^7^, ^8^ Similarly, an EORTC trial in 1997 showed that chemoradiation improved colostomy free survival, local regional control, and complete responses compared to radiation alone.^9^ Current National Cancer Center Network (NCCN) guidelines now recommend concurrent chemoradiation for stage I–III anal cancer.^9^

Racial disparities in receipt of standard therapy has been shown in other cancers. A 2014 study using the California cancer registry reported that Black race, low socioeconomic status, and longer distance from a high‐volume hospital were independently associated with an increased risk of care that did not adhere to NCCN guidelines in advanced ovarian cancer.^10^ Similarly, an NCDB study from 2016 showed that for localized medullary thyroid cancer, Black race, older age, lower median income, and treatment in a community center were associated with a lower likelihood of guideline adherent care.^11^ A Surveillance, Epidemiology, and End Results Program (SEER) study from 2016 also showed that Black race was an independent predictor of not receiving radiation plus androgen deprivation therapy, the standard of care in high‐risk prostate cancer.^12^ In anal cancer, one SEER study by Arora et al. showed that the rate of receipt of radiation therapy was lowest in Black men (77%) compared to the population overall (82%).^13^ Similarly, our study shows that Black patients had a 15% lower chance of receiving standard therapy compared to White patients.

There is conflicting data in the literature regarding racial disparities in anal SCC. Although some reports suggest racial disparities in anal SCC incidence and survival,^14^, ^15^ no studies have specifically investigated whether racial and ethnic minorities are less likely to receive standard therapy for anal SCC. We bridged this knowledge gap by studying racial disparities in standard therapy for SCC and whether disparities in standard treatment were associated with survival differences.

## 2 METHODS

### 2.1 Data source and population

We used the NCDB, a national cancer registry created and maintained by the American College of Surgeons’ Commission on Cancer (COC) and the American Cancer Society. It includes hospital registry data that are collected in more than 1500 COC‐accredited facilities, representing more than 70% of newly diagnosed cancer cases nationwide and more than 34 million historical records.

**FIGURE 1:**
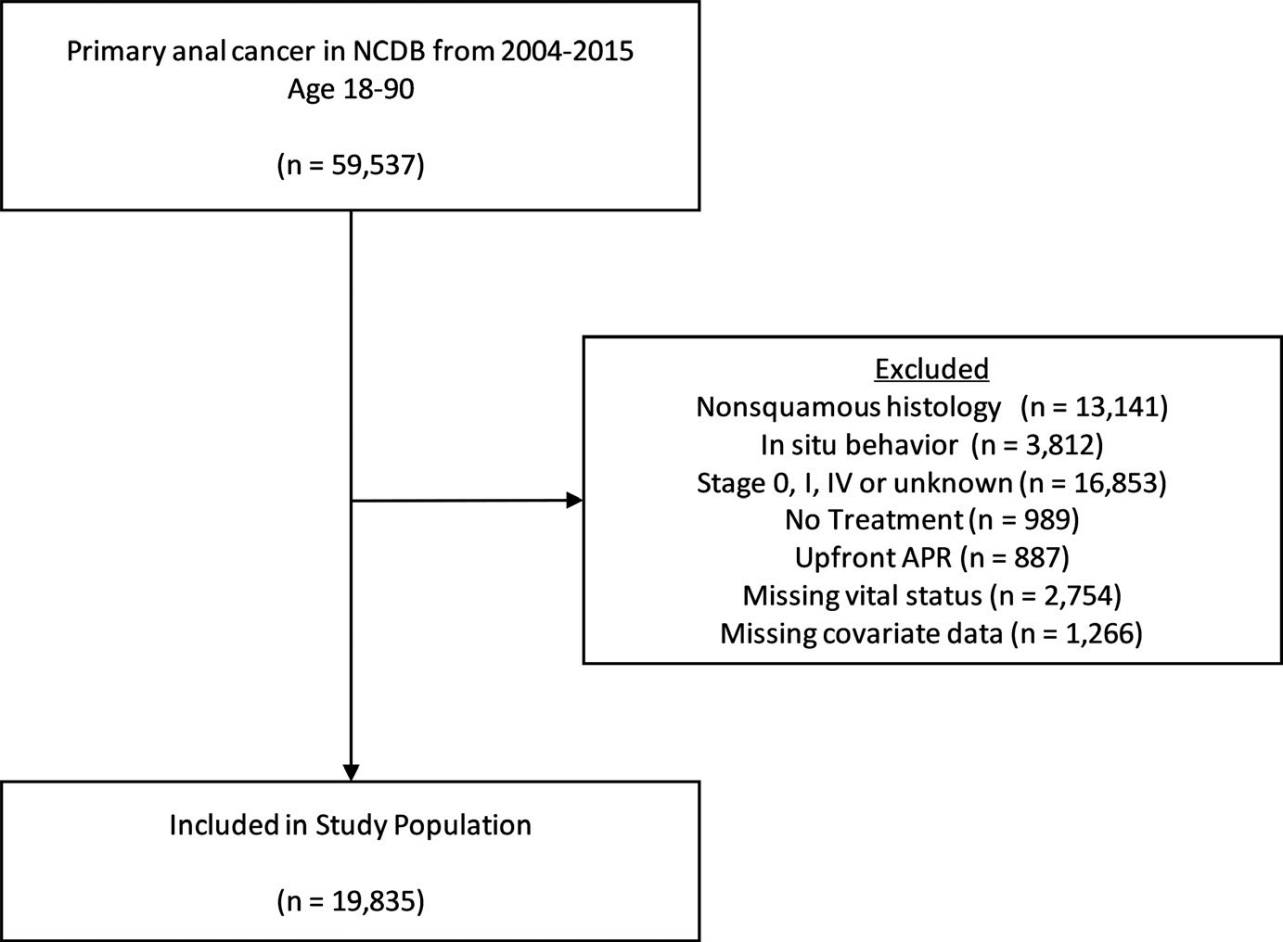
Consolidated Standards of Reporting Trials (CONSORT) Diagram

We identified patients aged 18–90 years with a diagnosis of anal cancer from 2004 to 2015. We limited our study population to patients with squamous cell carcinoma (SCC) histology, malignant tumor behavior, and stage II or III disease based on American Joint Committee on Cancer (AJCC) sixth or seventh edition cancer staging. We excluded patients with stage I disease because there is some controversy over the optimal treatment, with studies supporting excellent outcomes with local excision, radiation alone, in addition to concurrent chemoradiation.^16^, ^17^, ^18^, ^19^ Additionally, the randomized trials showing superiority of chemoradiation to radiation alone excluded stage I patients.^7^, ^8^, ^9^ We excluded stage IV patients because standard treatments in metastatic patients vary widely. We also excluded patients who received an APR as initial treatment and those with missing follow‐up. We present a CONSORT diagram of our inclusion/exclusion criteria in Figure 1.

### 2.2 Variables

We defined standard treatment as concurrent chemoradiation, or the receipt of chemotherapy and radiation therapy within 7 days of each other. We defined all other treatments as nonstandard, including nonconcurrent chemoradiation (receipt of chemotherapy and radiation therapy more than 7 days apart), local excision alone, local excision with any other therapy (including nonconcurrent chemoradiation, chemotherapy, or radiation therapy), chemotherapy alone, and radiation therapy alone. We studied patients’ overall survival and right‐censored patients if they were lost to follow‐up. Patients were followed for a maximum of 10 years.

Our main exposure was race (White, Black, and Other). Additionally, we controlled for sociodemographic (age, sex, ethnicity, and insurance status), biologic (stage, grade, and Charlson–Deyo comorbidity index), facility (academic vs. community and facility location), geographic (mean income and education of zip code, and whether the zip code was urban or rural), and temporal (year of diagnosis) characteristics. We show detailed specifications of covariates in Table 1.

**TABLE 1:** Patient, facility/demographic, and disease/treatment characteristics for standard versus nonstandard therapy in anal SCC

	Standard therapy (N = 15,332)	Nonstandard therapy (N = 4503)	*p*‐value
Patient characteristics			
Age			<0.01
<50	2657 (17.3)	762 (16.9)	
50–59	5376 (35.1)	1372 (30.5)	
60–69	4130 (26.9)	1008 (22.4)	
70+	3169 (20.7)	1361 (30.2)	
Sex			<0.01
Male	4615 (30.1)	1611 (35.8)	
Female	10,717 (69.9)	2892 (64.2)	
Race			<0.01
White	13,569 (88.5)	3867 (85.9)	
Black	1433 (9.3)	503 (11.2)	
Other	330 (2.2)	133 (3)	
Ethnicity			<0.01
Non‐Hispanic	13,868 (90.5)	3986 (88.5)	
Hispanic	1464 (9.5)	517 (11.5)	
Charlson–Deyo comorbidity index			<0.01
0	12,435 (81.1)	3522 (78.2)	
1	1927 (12.6)	613 (13.6)	
2–3	970 (6.3)	368 (8.2)	
Facility/demographic characteristics			
Facility type			<0.01
Community	1531 (10.0)	529 (11.7)	
Comprehensive Community	6956 (45.4)	2009 (44.6)	
Academic/research	5144 (33.6)	1521 (33.8)	
Integrated network	1701 (11.1)	444 (9.9)	
Facility location			<0.01
New England	920 (6.0)	219 (4.9)	
Middle Atlantic	2170 (14.2)	759 (16.9)	
South Atlantic	3619 (23.6)	962 (21.4)	
East North Central	2786 (18.2)	682 (15.1)	
East South Central	1002 (6.5)	376 (8.3)	
West North Central	1256 (8.2)	256 (5.7)	
West South Central	1024 (6.7)	364 (8.1)	
Mountain	696 (4.5)	170 (3.8)	
Pacific	1859 (12.1)	715 (15.9)	
Insurance			<0.01
Uninsured	918 (6)	248 (5.5)	
Private	6935 (45.2)	1679 (37.3)	
Public	7121 (46.4)	2469 (54.8)	
Unknown	358 (2.3)	107 (2.4)	
Median Income			<0.01
<$38,000	2951 (19.2)	945 (21.0)	
$38,000–$62,999	7932 (51.7)	2180 (48.4)	
$63,000+	4449 (29.0)	1378 (30.6)	
% Without high school degree			<0.01
>13%	6692 (43.6)	2166 (48.1)	
<=13%	8640 (56.4)	2337 (51.9)	
Residence			<0.01
Metropolitan	12,916 (84.2)	3919 (87.0)	
Urban	2160 (14.1)	518 (11.5)	
Rural	256 (1.7)	66 (1.5)	
Year of diagnosis			<0.01
2004–2010	6069 (39.6)	2235 (49.6)	
2011–2015	9263 (60.4)	2268 (50.4)	
Disease/treatment characteristics			
Stage			<0.01
2	8677 (56.6)	2711 (60.2)	
3	6655 (43.4)	1792 (39.8)	
Grade			<0.01
Well differentiated	1135 (7.4)	485 (10.8)	
Moderately differentiated	5568 (36.3)	1637 (36.4)	
Poorly/un‐differentiated	4370 (28.5)	1193 (26.5)	
Unknown	4259 (27.8)	1188 (26.4)	
Radiation dose – primary + boost			<0.01
<30 Gy	415 (2.7)	244 (5.4)	
30–40 Gy	508 (3.3)	213 (4.7)	
40–50 Gy	1702 (11.1)	461 (10.2)	
50–60 Gy	10,372 (67.6)	1963 (43.6)	
>60 Gy	1605 (10.5)	487 (10.8)	
None	0	891 (19.8)	
Unknown	730 (4.8)	244 (5.4)	
Radiation technique			<0.01
No IMRT	8729 (56.9)	3411 (75.7)	
IMRT	6603 (43.1)	1092 (24.3)	

Note

*p*‐values computed using Pearson chi‐square.

Abbreviations: IMRT, intensity‐modulated radiation therapy; SCC, squamous cell carcinoma.

### 2.3 Statistical analyses

In unadjusted analysis, we computed descriptive statistics of patient, facility, disease, and treatment characteristics between standard and nonstandard treatment groups, testing for statistical differences using a Pearson Chi‐Squared test. Using Kaplan–Meier plots, we examined the unadjusted effect of standard and nonstandard treatment on survival in the population as a whole and by stage. We conducted log‐rank tests to assess whether unadjusted survival differences were statistically significant.

We used multivariable logistic regression to assess for racial differences in standard therapy, controlling for covariates. To estimate differences in survival associated with standard therapy, we used a multivariable Cox proportional hazards model. In a subgroup analysis, we investigated racial differences in survival within the standard therapy and nonstandard therapy subgroups. We conducted subgroup analyses by including interaction terms between the race and standard therapy variables. To obtain subgroup‐specific hazard ratios, we exponentiated the sum of the relevant coefficients. We computed 95% confidence intervals using the delta method.

We used robust standard errors for all multivariable analyses, and all significance tests were two‐tailed, with *α* = 0.05. All analyses were performed using SAS software v. 9.4 (SAS Institute Inc.) and STATA v. 14.

### 2.4 Sensitivity analyses

To explore the robustness of our results to model specification, we conducted sensitivity analyses using an inverse probability of treatment weighting method, which is analytically similar to propensity score matching techniques. Given that these analyses did not deviate from our findings from our a priori specified primary analyses, we chose to present these as sensitivity analyses (Appendix).

## 3 RESULTS

We identified 19,835 patients that met our inclusion criteria. Median follow‐up was 41 months (interquartile range 21–70 months). Patient, facility, disease, and treatment characteristics for both standard and nonstandard treatment are presented in Table 1.

Patients receiving standard treatment were more likely younger, female, White, and non‐Hispanic, and more likely to have a lower Charlson–Deyo Comorbidity Index. They also were more likely to receive treatment in a comprehensive community, academic, or integrated network facility, to have private insurance, to reside in a nonmetropolitan area, to reside in a zip code with higher median income and more high school graduates, and to have a more recent diagnosis date. Patients with stage III disease and poorly or undifferentiated tumors were more likely to receive standard therapy. About 20% of patients in the nonstandard therapy group did not receive radiation therapy. The majority of patients in both groups who did receive radiation had doses of at least 40 Gy. Additionally, patients receiving standard therapy were more likely to receive Intensity Modulated Radiation Therapy (IMRT) planning. This newer radiation technique is more conformal and has been shown to decrease treatment‐related toxicity.^20^

Table 2 shows the distribution of treatment regimens by stage. Most patients received standard therapy, 76.2% in stage II and 78.8% in stage III. The most common nonstandard therapy was nonconcurrent chemotherapy and radiation therapy without surgery, (7.1% in stage II and 9.4% in stage III), followed by radiation alone (6.1% in stage II and III). Other treatment combinations made up less than 10% of the population.

**TABLE 2:** Treatment by stage for anal SCC

Treatment	Stage II	Stage III	Total
	N (%)	N (%)	N (%)
Chemo/RT within 7 days	8677 (76.2)	6655 (78.8)	15,332 (77.3)
Nonstandard Chemo/RT without surgery	808 (7.1)	794 (9.4)	1602 (8.1)
Local excision without Chemo or RT	573 (5.0)	94 (1.1)	667 (3.4)
Local excision with Chemo or RT	528 (4.6)	241 (2.9)	769 (3.9)
Chemo alone	112 (1.0)	145 (1.7)	257 (1.3)
RT alone	690 (6.1)	518 (6.1)	1208 (6.1)
Total	11,388	8447	19,835

Abbreviations: RT, radiation therapy.

### 3.1 Racial disparities in standard treatment

On multivariable analysis (Table 3), Black patients and patients of Other races were less likely to receive standard treatment compared to White patients (OR 0.85, 95%CI 0.76–0.96; *p* < 0.008, and OR 0.78, 95%CI 0.63–0.97; *p* < 0.02, respectively).

**TABLE 3:** Multivariable analysis ‐ likelihood of receiving standard therapy in anal SCC

Covariate	Odds ratio (95% CI)	*p*‐value
Age		
<50	Reference	
50–59	1.01 (0.91–1.12)	0.84
60–69	1.07 (0.96–1.20)	0.21
70+	0.64 (0.57–0.72)	0.001
Sex		
Male	Reference	
Female	1.23 (1.14–1.32)	0.001
Race		
White	Reference	
Black	0.85 (0.76–0.96)	0.008
Other	0.78 (0.63–0.97)	0.02
Ethnicity		
Non‐Hispanic	Reference	
Hispanic	0.87 (0.78–0.97)	0.02
Charlson–Deyo comorbidity index		
0	Reference	
1	0.93 (0.84–1.03)	0.14
2–3	0.83 (0.73–0.95)	0.006
Facility/demographic characteristics		
Facility type		
Community	0.81 (0.72–0.91)	0.001
Comprehensive Community	1.01 (0.93–1.10)	0.76
Academic/Research	Reference	
Integrated Network	1.10 (0.97–1.24)	0.15
Facility location		
New England	Reference	
Middle Atlantic	0.68 (0.57–0.81)	0.001
South Atlantic	0.86 (0.73–1.02)	0.09
East North Central	0.93 (0.78–1.11)	0.42
East South Central	0.59 (0.49–0.72)	0.001
West North Central	1.05 (0.85–1.28)	0.67
West South Central	0.66 (0.54–0.80)	0.001
Mountain	0.86 (0.68–1.08)	0.19
Pacific	0.60 (0.51–0.72)	0.001
Insurance		
Uninsured	1.07 (0.92–1.26)	0.37
Private	1.19 (1.09–1.29)	0.001
Public	Reference	
Unknown	1.06 (0.84–1.33)	0.65
Median income		
<$38,000	Reference	
$38,000–$62,999	1.05 (0.95–1.15)	0.36
$63,000+	0.85 (0.76–0.97)	0.01
% Without high school degree		
>13%	Reference	
<=13%	1.20 (1.10–1.30)	0.001
Residence		
Metropolitan	Reference	
Urban	1.23 (1.10–1.38)	0.001
Rural	1.20 (0.90–1.59)	0.22
Year of diagnosis		
2004–2010	Reference	
2011–2015	1.49 (1.39–1.60)	0.001
Disease/treatment characteristics		
Stage		
2	Reference	
3	1.09 (1.02–1.17)	0.01
Grade		
Well differentiated	Reference	
Moderately differentiated	1.39 (1.23–1.57)	0.001
Poorly/un‐differentiated	1.48 (1.31–1.69)	0.001
Unknown	1.47 (1.29–1.67)	0.001

Note

Estimated using logistic regression.

Abbreviations: CI, confidence interval; SCC, squamous cell carcinoma.

### 3.2 Standard treatment as a predictor for survival

**FIGURE 2:**
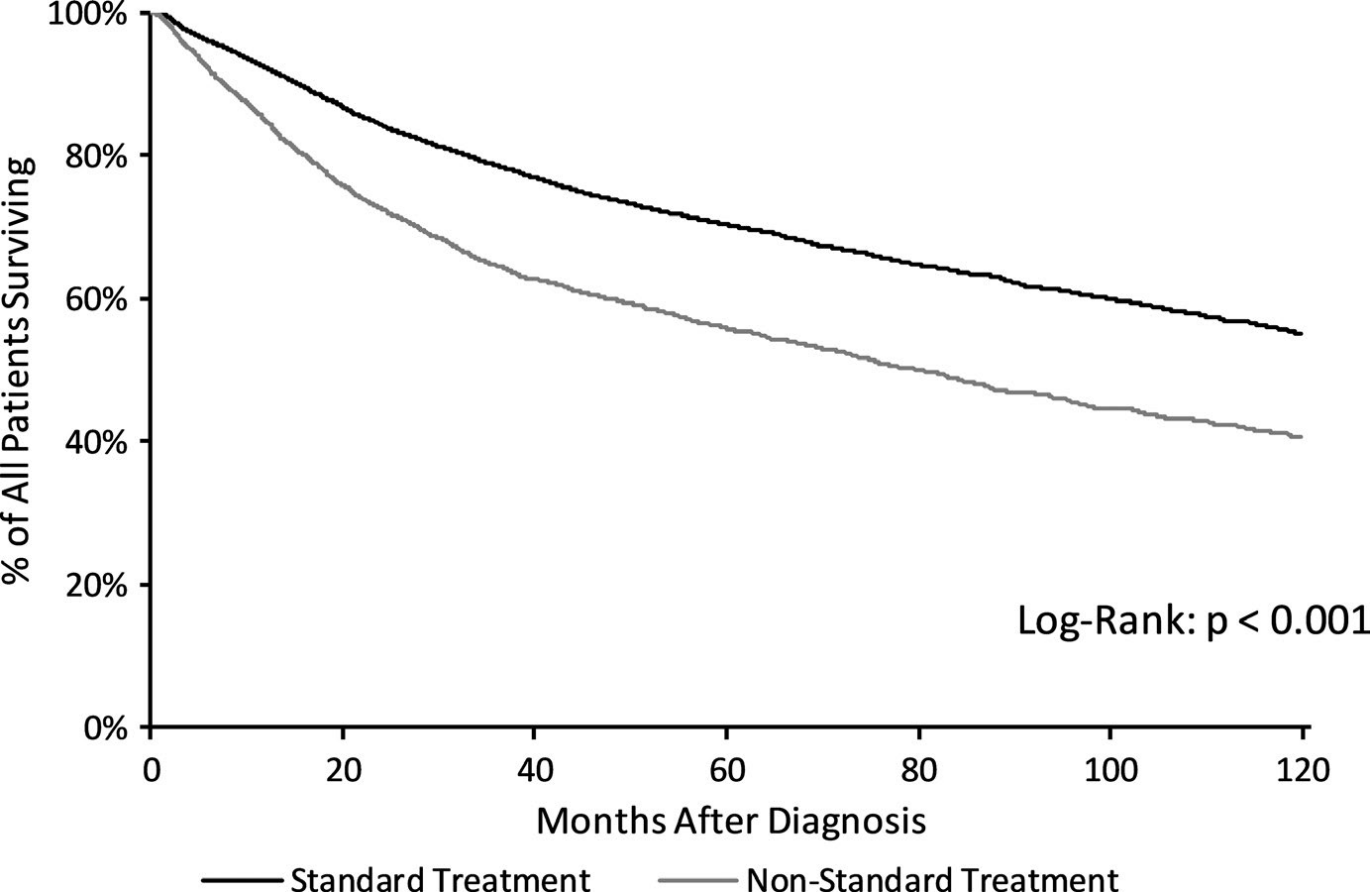
Kaplan–Meier ‐ survival in standard versus nonstandard treatment for stage II–III Anal SCC. *p*‐value computed using Log‐rank test. Abbreviations: SCC, Squamous Cell Carcinoma

In unadjusted analysis, standard treatment was associated with higher rates of survival compared to nonstandard treatment (Figure 2), with separation starting at the time of diagnosis and persisting through the end of our follow‐up period of 10 years. Survival for standard treatment versus nonstandard treatment were 70.4% versus 55.9% at 5 years and 55.3% versus 40.6% at 10 years, respectively.

**FIGURE 3:**
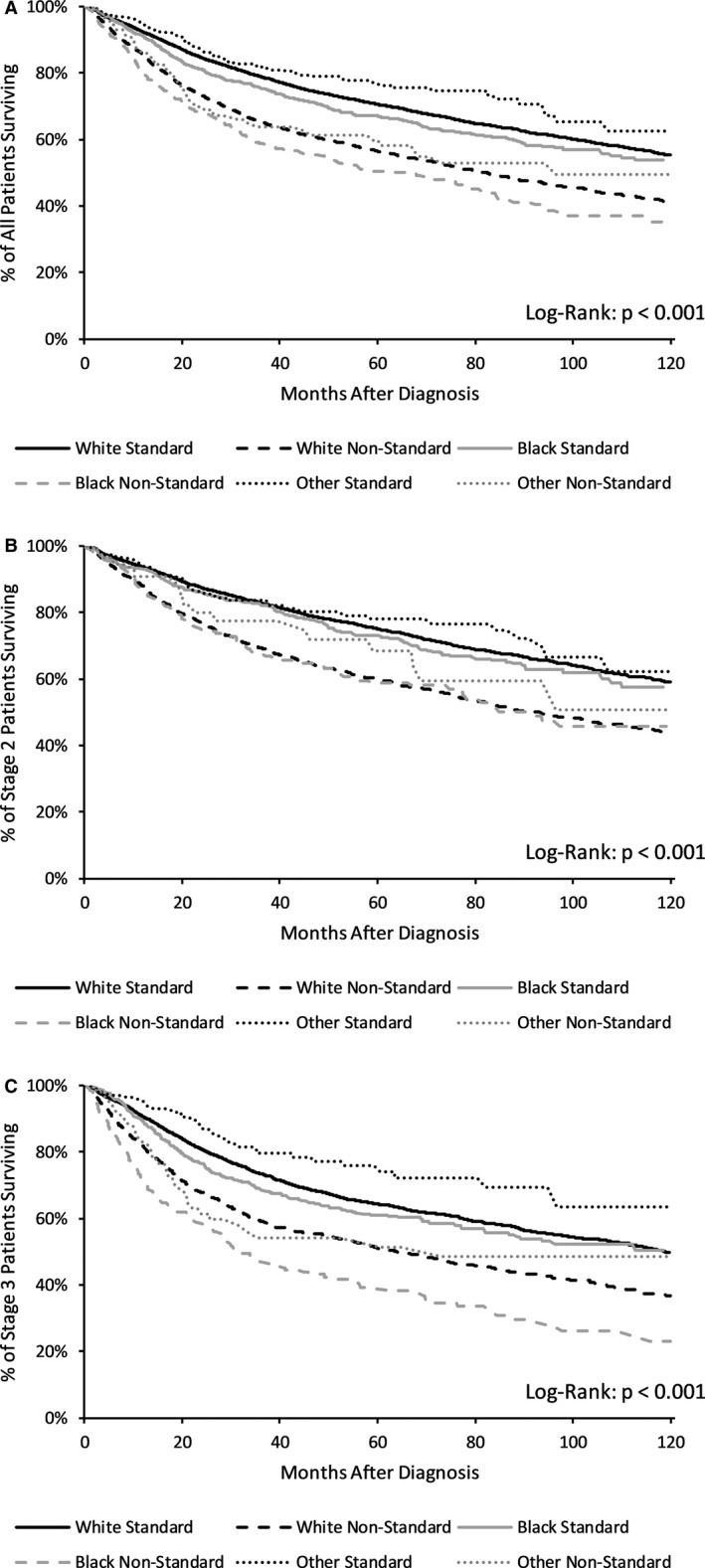
Kaplan–Meier ‐ survival in standard versus nonstandard treatment for anal SCC by race in (A) All patients, (B) Stage II, and (C) Stage III. *p*‐values computed using Log‐rank test. Abbreviations: SCC, Squamous Cell Carcinoma

Among patients who received standard therapy, 5‐year survival was 70.6% versus 67.0% and 10‐year survival was 55.3% versus 52.7% for White and Black patients, respectively. For patients who received nonstandard therapy, 5‐year survival was 56.5% versus 50.5% and 10‐year survival was 41.0% versus 35.0% for White versus Black patients, respectively. Additional 5‐ and 10‐year survival rates by stage can be found in Table 4. Racial differences in survival between standard and nonstandard treatment were most pronounced in patients with Stage III disease (Figure 3).

**TABLE 4:** Survival by race and stage in anal SCC ‐ 5‐ and 10‐year rates

Group	5‐Year survival	10‐year survival
Standard all	70.4%	55.3%
White standard all	70.6%	55.3%
Black standard all	67.1%	53.7%
Other standard all	76.3%	62.4%
Nonstandard all	55.9%	40.6%
White nonstandard all	56.5%	41.1%
Black nonstandard all	50.5%	35.1%
Other nonstandard all	59.8%	49.5%
Standard stage II	74.9%	60.0%
White standard stage II	75.0%	59.1%
Black standard stage II	73.3%	57.5%
Other standard stage II	78.0%	62.3%
Nonstandard stage II	60.0%	43.9%
White nonstandard stage II	60.0%	43.7%
Black nonstandard stage II	58.8%	45.6%
Other nonstandard stage II	68.5%	50.9%
Standard stage III	64.2%	50.2%
White standard stage III	64.3%	49.9%
Black standard stage III	61.1%	50.5%
Other standard stage III	74.1%	63.6%
Nonstandard stage III	49.6%	35.3%
White nonstandard stage III	50.9%	36.7%
Black nonstandard stage III	38.4%	23.0%
Other nonstandard stage III	51.4%	48.5%

Abbreviations: SCC, squamous cell carcinoma.

On multivariable Cox regression, after adjusting for covariates, standard concurrent chemoradiation was associated with a lower probability of death relative to nonstandard treatment (HR 0.64; 95% CI 0.61–0.68; *p* < 0.001) (Table 5). In the entire population, we did not observe statistically significant survival differences in Black race (HR 1.05, 95%CI 0.97–1.15; *p* = 0.24) or patients of Other race (HR 0.85, 95%CI 0.70–1.03; *p* = 0.10), compared to White race.

**TABLE 5:** Multivariable analysis ‐ predictors of survival in anal SCC

Covariate	Hazard ratio (95% CI)	*p*‐value
Treatment		
Standard	0.64 (0.61–0.68)	0.001
Nonstandard	Reference	
Patient characteristics		
Age		
<50	Reference	
50–59	1.14 (1.05–1.24)	0.002
60–69	1.32 (1.21–1.44)	0.001
70+	2.24 (2.05–2.44)	0.001
Sex		
Male	Reference	
Female	0.66 (0.62–0.69)	0.001
Race		
White	Reference	
Black	1.05 (0.97–1.15)	0.24
Other	0.85 (0.70–1.03)	0.10
Ethnicity		
Non‐Hispanic	Reference	
Hispanic	0.88 (0.80–0.96)	0.003
Charlson–Deyo comorbidity index		
0	Reference	
1	1.41 (1.32–1.52)	0.001
2–3	1.92 (1.76–2.10)	0.001
Facility/demographic characteristics		
Facility type		
Community	1.21 (1.10–1.32)	0.001
Comprehensive Community	1.13 (1.06–1.20)	0.001
Academic/Research	Reference	
Integrated Network	1.07 (0.97–1.17)	0.18
Facility location		
New England	Reference	
Middle Atlantic	1.04 (0.92–1.18)	0.54
South Atlantic	1.06 (0.94–1.19)	0.36
East North Central	1.12 (0.99–1.26)	0.07
East South Central	1.05 (0.91–1.21)	0.50
West North Central	1.03 (0.90–1.18)	0.68
West South Central	1.03 (0.89–1.19)	0.71
Mountain	1.04 (0.89–1.22)	0.64
Pacific	0.99 (0.88–1.13)	0.91
Insurance		
Uninsured	0.90 (0.80–1.01)	0.07
Private	0.62 (0.58–0.66)	0.001
Public	Reference	
Unknown	0.70 (0.58–0.85)	0.001
Median income		
<$38,000	Reference	
$38,000–$62,999	0.97 (0.91–1.05)	0.46
$63,000+	0.86 (0.78–0.94)	0.002
% Without high school degree		
>13%	Reference	
<=13%	0.96 (0.90–1.02)	0.18
Residence		
Metropolitan	Reference	
Urban	0.97 (0.90–1.05)	0.44
Rural	1.13 (0.94–1.37)	0.21
Year of diagnosis		
2004–2010	Reference	
2011–2015	0.91 (0.86–0.96)	0.001
Disease/treatment characteristics		
Stage		
2	Reference	
3	1.62 (1.54–1.70)	0.001
Grade		
Well differentiated	Reference	
Moderately differentiated	1.06 (0.96–1.17)	0.25
Poorly/un‐differentiated	1.01 (0.91–1.11)	0.92
Unknown	0.99 (0.89–1.09)	0.81

Note

Estimated using Cox proportional hazards model.

Abbreviations: CI, confidence interval; SCC, squamous cell carcinoma.

However, we found racial differences within the subgroup of patients receiving nonstandard therapy. Black patients had significantly higher rates of death compared to White patients (HR 1.17, CI 1.01–1.35; *p* = 0.034). In patients of Other races, there was no significant difference in survival compared to White patients (HR 0.86, CI 0.62–1.18; *p* = 0.34). Conversely, within the standard therapy subgroup, we observed no statistical differences in survival between Black and White patients (HR 1.00, CI 0.90–1.11, *p* = 0.99) or between patients of Other race and White patients (HR 0.85, CI 0.67–1.08, *p* = 0.18). Results of this subgroup analysis are shown in Table 6.

**TABLE 6:** Subgroup multivariable analysis – survival in standard versus nonstandard therapy by race

	Hazard ratio (95%CI)	*p*‐value
Standard therapy		
Black versus White Race	1.00 (0.90–1.11)	0.99
Other versus White Race	0.85 (0.67–1.08)	0.18
Nonstandard therapy		
Black versus White Race	1.17 (1.01–1.35)	0.03
Other versus White Race	0.86 (0.62–1.18)	0.34

Note

Estimated using Cox Proportional Hazards model with interactional terms between race and receipt of standard therapy, adjusting for the patient level (demographic and disease/treatment), facility level, geographic level (zip code socioeconomics), and temporal (year of diagnosis) characteristics in Table 1. Subgroup‐specific hazard ratios estimated by exponentiating the linear combination of coefficients, with standard errors calculated using the delta method.

Abbreviation: CI, confidence interval.

Findings were not materially different in sensitivity analyses (Appendix Tables 1–2).

## 4 DISCUSSION

In this study of patients with stage II–III anal SCC from the NCDB database, we found that receipt of standard therapy, as defined by concurrent chemoradiation therapy, was associated with improved survival after controlling for other covariates. We identified substantial racial disparities in receipt of standard therapy, with Black patients significantly less likely to receive standard therapy compared to White patients. Racial differences in standard therapy were associated with material differences in survival. Black patients receiving nonstandard therapy had a 17% higher probability of death than White patients receiving nonstandard therapy. Racial differences in mortality was not present among patients who received standard, NCCN guideline‐concordant therapy.

In anal cancer, several studies suggest disparities in outcomes across different facility types. Bitterman et al. observed that patients referred from public hospitals experienced worse survival and significantly longer radiotherapy delays and duration compared to those referred from private hospitals.^21^ Using data from the National Cancer Database (NCDB), Amini et al. found that SCC patients treated at high‐volume cancer centers experienced better OS and fewer treatment delays than patients treated at low‐volume cancer centers.^22^ While the cause of these disparities is likely multifactorial, variability in the receipt of standard concurrent chemoradiation therapy is likely one of the main drivers.

The literature has had conflicting results on the presence of racial disparities on survival in anal cancer. An older NCDB study from 1985 to 2000 found that Black race was independently associated with worse survival.^14^ Similarly, two SEER analyses, one from 2000 to 2012 and one from 2000 to 2013 both showed lower survival for Black patients after controlling for sex, age, stage, grade, surgery, and radiation therapy.^13^, ^23^ The authors hypothesized that this racial disparity could be due to an interplay of structural, cultural, and social barriers to healthcare as well as tumor biology. On the contrary, two more recent NCDB studies, one from 2004 to 2013 and one from 2004 to 2014 both did not find evidence of racial disparities in survival on multivariable analysis.^22^, ^24^

Our study potentially reconciles this conflict in findings by suggesting that racial disparities in treatment is a plausible mechanism for survival differences. Like the previous NCDB studies, we also did not observe survival differences associated with race overall. However, among patients receiving nonstandard therapy, Black patients had significantly higher mortality than White patients. A potential explanation unifying the findings of previous studies is the improvement of adherence to standard therapy over time, such that racial disparities in survival are no longer readily apparent when examining the cohort as a whole. Indeed, we found a 50% increase in the odds of receiving standard therapy between 2011 and 2014, relative to 2004–2010. The studies demonstrating racial disparities included patients prior to 2004, while those showing no differences among racial groups included a more recent cohort. Haque et al. corroborates these results with a 2018 NCDB analysis, showing that IMRT usage has increased significantly from 28% in 2004 to 96% in 2015, indicating a national transition toward a more modern, standardized approach to treatment.

Improvements in the use of standard therapy notwithstanding, Black patients continue to lag behind White patients. Although a majority of patients now receive standard therapy, our findings suggest that differences likely contribute to racial differences in survival. Prioritizing guideline adherent treatment on the institutional level, particularly among safety‐net hospitals and providers, could either reduce or eliminate these disparities altogether.

There are important limitations to our study. The retrospective nature of the NCDB means that the analysis is subject to potential coding and clerical errors. As with other observational studies using administrative databases, our results could be biased by residual confounding from unobserved patient and facility characteristics. Our study can only describe associations and although it is suggestive of putative mechanisms for racial disparities in survival, it does not provide causal evidence. Furthermore, hospitals reporting to the NCDB must be COC approved, which may limit generalizability and skew the data set toward centers with higher levels of cancer specialization. We conjecture, however, that this would likely lead to an underestimation of racial disparities since lower‐funded and less‐specialized treatment centers taking care of patients of lower socioeconomic status may be excluded.

Strengths of our study include using an updated national data set that captures detailed treatment patterns over a modern period. The data set captures patient and facility characteristics across biologic and sociodemographic domains, reducing the risk of bias. Unlike previous studies, we explored potential mechanisms for racial disparities and provide evidence that increasing standard therapy among patients with anal SCC could alleviate or eliminate racial disparities in survival.

## 5 CONCLUSION

Standard concurrent chemoradiation in anal SCC was associated with better overall survival compared to other treatment regimens. Black patients were less likely to receive standard treatment than their White counterparts. Although Black patients receiving nonstandard therapy had higher rates of mortality than White patients, this disparity was ameliorated when receiving standard therapy. Increasing physician awareness of and adherence to standard treatment recommendations could potentially improve these racial disparities.

## CONFLICTS OF INTEREST

None.

## AUTHOR CONTRIBUTIONS

Shelly X. Bian: Conceptualization, methodology, resources, writing‐original draft, writing‐review and editing, supervision. Dennis Chen: Conceptualization, methodology, writing‐review and editing. Eugene Lin: Conceptualization, methodology, formal analysis, writing‐review and editing.

## Supporting information

Appendix

## Data Availability

All data used in this publication are publicly available through the National Cancer Database.

## References

[1] Siegel RL , Miller KD , Jemal A . Cancer statistics, 2019. CA Cancer J Clin. 2019;69(1):7‐34. 10.3322/caac.21551 30620402

[2] Hoff PM , Coudry R , Motta C , Moniz V . Pathology of anal cancer. Surg Oncol Clin NA. 2017;26(1):57‐71. 10.1016/j.soc.2016.07.013 27889037

[3] Johnson LG , Madeleine MM , Newcomer LM , Schwartz SM , Daling JR . Anal cancer incidence and survival: the Surveillance, Epidemiology, and End Results experience, 1973–2000. Cancer. 2004;101(2):281‐288. 10.1002/cncr.20364 15241824

[4] Nelson RA , Levine AM , Bernstein L , Smith DD , Lai LL . Changing patterns of anal canal carcinoma in the United States. J Clin Oncol. 2013;31(12):1569‐1575. 10.1200/JCO.2012.45.2524 23509304 PMC3753461

[5] Nigro ND , Seydel HG , Considine B , Vaitkevicius VK , Leichman L , Kinzie JJ . Combined preoperative radiation and chemotherapy for squamous cell carcinoma of the anal canal. Cancer. 1983;51(10):1826‐1829. 10.1002/1097-0142(19830515)51:10<1826:AID-CNCR2820511012>3.0.CO;2-L 6831348

[6] Shridhar R , Shibata D , Chan E , Thomas CR . Anal cancer: current standards in care and recent changes in practice. CA Cancer J Clin. 2015;65(2):139‐162. 10.3322/caac.21259 25582527

[7] Northover JMA , Arnott SJ , Cunningham D , et al. Epidermoid anal cancer: results from the UKCCCR randomised trial of radiotherapy alone versus radiotherapy, 5‐fluorouracil, and mitomycin. Lancet. 1996;348(9034):1049‐1054. 10.1016/S0140-6736(96)03409-5 8874455

[8] Northover J , Glynne‐Jones R , Sebag‐Montefiore D , et al. Chemoradiation for the treatment of epidermoid anal cancer: 13‐year follow‐up of the first randomised UKCCCR Anal Cancer Trial (ACT I). Br J Cancer. 2010;102(7):1123‐1128. 10.1038/sj.bjc.6605605 20354531 PMC2853094

[9] Bartelink H , Roelofsen F , Eschwege F , et al. Concomitant radiotherapy and chemotherapy is superior to radiotherapy alone in the treatment of locally advanced anal cancer: results of a phase III randomized trial of the European organization for research and treatment of cancer radiotherapy and gastrointestinal cooperative groups. J Clin Oncol. 1997;15(5):2040‐2049. 10.1200/JCO.1997.15.5.2040 9164216

[10] Bristow RE , Chang J , Ziogas A , Anton‐Culver H , Vieira VM . Spatial analysis of adherence to treatment guidelines for advanced‐stage ovarian cancer and the impact of race and socioeconomic status. Gynecol Oncol. 2014;134(1):60‐67. 10.1016/j.ygyno.2014.03.561 24680770 PMC4095874

[11] Chang EHE , Lutfi W , Feinglass J , Reiher AE , Moo‐Young T , Bhayani MK . National trends in the surgical treatment of non‐advanced Medullary Thyroid Cancer (MTC): an evaluation of adherence with the 2009 American Thyroid Association guidelines. World J Surg. 2016;40(12):2930‐2940. 10.1007/s00268-016-3643-6 27447700

[12] Dell’Oglio P , Abou‐Haidar H , Leyh‐Bannurah SR , et al. Assessment of the rate of adherence to international guidelines for androgen deprivation therapy with external‐beam radiation therapy: a population‐based study. Eur Urol. 2016;70(3):429‐435. 10.1016/j.eururo.2016.02.057 26951945

[13] Arora N , Gupta A , Zhu H , et al. Race‐ and sex‐based disparities in the therapy and outcomes of squamous cell carcinoma of the anus. JNCCN J Natl Compr Cancer Netw. 2017;15(8):998‐1004. 10.6004/jnccn.2017.0135 28784861

[14] Bilimoria KY , Bentrem DJ , Rock CE , Stewart AK , Ko CY , Halverson A . Outcomes and prognostic factors for squamous‐cell carcinoma of the anal canal: analysis of patients from the national cancer data base. Dis Colon Rectum. 2009;52(4):624‐631. 10.1007/DCR.0b013e31819eb7f0 19404066

[15] Cruz A , Chen D , Hsu P , et al. Racial and gender disparities in the incidence of anal cancer: analysis of the Nationwide Inpatient Sample (NIS). J Gastrointest Oncol. 2019;10(1):37‐41. 10.21037/jgo.2018.10.09 30788157 PMC6351293

[16] Chai CY , Cao HT , Awad S , Massarweh NN . Management of stage I squamous cell carcinoma of the anal canal. JAMA Surg. 2018;153(3):209‐215. 10.1001/jamasurg.2017.3151 29049547 PMC5885927

[17] Touboul E , Schlienger M , Buffat L , et al. Epidermoid carcinoma of the anal canal. Results of curative‐intent radiation therapy in a series of 270 patients. Cancer. 1994;73(6):1569‐1579. 10.1002/1097-0142(19940315)73:6<1569:AID-CNCR2820730607>3.0.CO;2-F 8156483

[18] Newman G , Calverley DC , Acker BD , Manji M , Haya J , Flores AD . The management of carcinoma of the anal canal by external beam radiotherapy, experience in Vancouver 1971–1988. Radiother Oncol. 1992;25(3):196‐202. 10.1016/0167-8140(92)90268-Y 1470696

[19] Ortholan C , Ramaioli A , Peiffert D , et al. Anal canal carcinoma: early‐stage tumors ≤10 mm (T1 or Tis): therapeutic options and original pattern of local failure after radiotherapy. Int J Radiat Oncol Biol Phys. 2005;62(2):479‐485. 10.1016/j.ijrobp.2004.09.060 15890590

[20] Kachnic LA , Winter K , Myerson RJ , et al. RTOG 0529: a phase 2 evaluation of dose‐painted intensity modulated radiation therapy in combination with 5‐fluorouracil and mitomycin‐C for the reduction of acute morbidity in carcinoma of the anal canal. Int J Radiat Oncol Biol Phys. 2013;86(1):27‐33. 10.1016/j.ijrobp.2012.09.023 23154075 PMC3619011

[21] Bitterman DS , Grew D , Gu P , et al. Comparison of anal cancer outcomes in public and private hospital patients treated at a single radiation oncology center. J Gastrointest Oncol. 2015;6(5):524‐533. 10.3978/j.issn.2078-6891.2015.061 26487947 PMC4570920

[22] Amini A , Jones BL , Ghosh D , Schefter TE , Goodman KA . Impact of facility volume on outcomes in patients with squamous cell carcinoma of the anal canal: analysis of the National Cancer Data Base. Cancer. 2017;123(2):228‐236. 10.1002/cncr.30327 27571233

[23] Bojko MM , Kucejko RJ , Poggio JL . Racial disparities and the effect of county level income on the incidence and survival of young men with anal cancer. Heal Equity. 2018;2(1):193‐198. 10.1089/heq.2018.0018 PMC6110184 30283867

[24] Ramey SJ , Rich BJ , Kwon D , et al. Demographic disparities in delay of definitive chemoradiation for anal squamous cell carcinoma: a nationwide analysis. J Gastrointest Oncol. 2018;9(6):1109‐1126. 10.21037/jgo.2018.08.07 30603130 PMC6286932

