# Supplementary material for: Racial disparities in receipt of standard chemoradiation in anal squamous cell carcinoma, an analysis of the National Cancer Database
Source: Cancer Med. 2020 Dec 11;10(2):575–85. doi: 10.1002/cam4.3625 (PMC7877367; doi:10.1002/cam4.3625)
Supplement: Supplementary file 1 — Appendix [file CAM4-10-575-s001.docx]

**Appendix for “****Racial Disparities in Receipt of Standard Chemoradiation in Anal Squamous Cell Carcinoma, an Analysis of the National Cancer Database”**

*Sensitivity Analyses, Methodology*

We used a propensity score method to assess the robustness of our results. We chose not to present this as our primary analysis for three reasons: results did not deviate from our *a priori* conceived primary analysis; the majority of patients received standard therapy; and we could not compute a propensity score for race since race is immutable. These exploratory sensitivity analyses corroborated our primary analyses.

In all analyses, we ran a Cox proportional hazards model with inverse probability of treatment weights, which we estimated using the propensity score^1^. The main advantage of IPTW over traditional matching methods is that it does not exclude data from the sample.

Our basic IPTW framework involved performing a logistic regression with the receipt of standard therapy as the dependent variable and all other covariates as the independent variable. From regression coefficients, we estimated the predicted probability of receiving standard therapy for each patient. We then estimated observation weights for each patient i:

$$w_{i}=\frac{Z_{i}}{e_{i}}+\frac{1-Z_{i}}{1-e_{i}}$$

where $w_{i}$ is the observation weight

$Z_{i}$ is whether the patient received standard therapy

$e_{i}$ is the predicted probability of receiving standard therapy (i.e., the propensity score)

Subsequently, we performed a Cox proportional hazards regression where we estimated the all-cause mortality rate as a function of receiving standard therapy. In the subgroup analysis where we excluded race from the propensity score model, the Cox proportional hazards model included receipt of standard therapy, race, and interaction terms as covariates. For the standard and non-standard therapy subgroups, we estimated the hazard ratios of race (Black versus White, and Other race versus White) by combining coefficients using the delta method. For both Cox models, we right censored for loss to follow-up.

For both analyses, we show raw and weighted standardized mean differences and variance ratios to demonstrate that our IPTW method achieved covariate balance. Standardized mean differences closer to 0 and variance ratios closer to 1 indicate better covariate balance.

*Sensitivity Analyses, Results*

In both models (including and excluding race from the propensity score), we saw substantial improvement in covariate balance with the IPTW method, with the absolute value of standardized differences ≤ 1.1% (**Appendix Table 1**). The distance between the weighted variance ratios and 1 were no more than 0.02 for all covariates.

Similar to our primary results, we observed that patients receiving standard therapy were significantly less likely to die (HR 0.67, 95% CI: [0.64, 0.71]) (**Appendix Table 2**).

**Appendix Table 1: Covariate Balance when Weighting by the Inverse Probability Treatment Weight, using All Covariates**

| **Characteristic** | | **Standardized Differences** | | **Variance Ratios** | |
| --- | --- | --- | --- | --- | --- |
|  |  | **Raw** | **Weighted** | **Raw** | **Weighted** |
| **Age** | **50-59** | -9.8% | 0.2% | 0.93 | 1.00 |
|  | **60-69** | -10.6% | -0.3% | 0.88 | 1.00 |
|  | **70+** | 22.1% | 0.6% | 1.29 | 1.01 |
| **Sex** | **Female** | -12.1% | -0.7% | 1.09 | 1.01 |
| **Race** | **Black** | 6.0% | 0.4% | 1.17 | 1.01 |
|  | **Other** | 5.1% | 0.2% | 1.36 | 1.02 |
| **Ethnicity** | **Hispanic** | 6.3% | 0.6% | 1.18 | 1.01 |
| **Grade** | **Moderately Differentiated** | 0.1% | -0.8% | 1.00 | 1.00 |
|  | **Poorly/Un- Differentiated** | -4.5% | 0.2% | 0.96 | 1.00 |
|  | **Unknown** | -3.1% | 0.9% | 0.97 | 1.01 |
| **Charlson** | **1** | 3.1% | 0.1% | 1.07 | 1.00 |
|  | **2+** | 7.1% | 0.6% | 1.27 | 1.02 |
| **Facility Type** | **Community** | 5.7% | -0.1% | 1.15 | 1.00 |
|  | **Comprehensive Community** | -1.5% | -0.6% | 1.00 | 1.00 |
|  | **Integrated Network** | -4.0% | 0.6% | 0.90 | 1.01 |
| **Facility Location** | **Middle Atlantic** | 7.5% | -0.1% | 1.15 | 1.00 |
|  | **South Atlantic** | -5.4% | 0.4% | 0.93 | 1.00 |
|  | **East North Central** | -8.1% | 0.5% | 0.86 | 1.01 |
|  | **East South Central** | 6.9% | -0.2% | 1.25 | 0.99 |
|  | **West North Central** | -9.9% | 0.3% | 0.71 | 1.01 |
|  | **West South Central** | 5.4% | -0.3% | 1.19 | 0.99 |
|  | **Mountain** | -3.8% | -0.5% | 0.84 | 0.98 |
|  | **Pacific** | 10.8% | -0.6% | 1.25 | 0.99 |
| **Insurance** | **Uninsured** | -2.1% | 0.3% | 0.92 | 1.01 |
|  | **Private** | -16.2% | -1.0% | 0.94 | 1.00 |
|  | **Unknown** | 0.3% | -0.2% | 1.02 | 0.99 |
| **Income** | **$38,000 - $62,999** | -6.6% | 0.6% | 1.00 | 1.00 |
|  | **$63,000+** | 3.5% | -0.7% | 1.03 | 0.99 |
| **High School Graduate** | **<= 13%** | -8.9% | -0.1% | 1.02 | 1.00 |
| **Metropolitan Size** | **Urban** | -7.7% | 1.1% | 0.84 | 1.02 |
|  | **Rural** | -1.6% | 0.3% | 0.88 | 1.02 |
| **Year of Diagnosis** | **2010-2015** | -20.3% | 0.3% | 1.05 | 1.00 |
| **Stage** | **Stage 3** | -7.3% | 1.1% | 0.98 | 1.00 |

Notes: Propensity scores estimated using receipt of standard treatment as the dependent variable and all listed covariates as independent variables. Weighted standardized differences closer to 0 and weighted variance ratios closer to 1 indicate better balance. Reference covariates omitted from table.

**Appendix Table 2: Associations between All-Cause Mortality and Receipt of Standard Therapy, using Inverse Probability Treatment Weights**

|  | **HR** | **95% CI** | **p-value** |
| --- | --- | --- | --- |
| **All Patients** | | | |
| Standard Therapy (vs. non-Standard Therapy) | 0.67 | 0.64, 0.71 | < 0.001 |

Notes: All estimates computed using a Cox proportional hazards model, associating all-cause mortality with receipt of standard therapy. Models were adjusted by using inverse probability treatment weights to weight observations. Weights were estimated from the propensity score, which adjusted for covariates.

Abbreviations: HR = hazard ratio, CI = confidence interval

**APPENDIX REFERENCES**

1. Austin PC. The use of propensity score methods with survival or time-to-event outcomes: Reporting measures of effect similar to those used in randomized experiments. *Stat Med*. 2014;33(7):1242-1258. doi:10.1002/sim.5984
